# Acute Seroconversion of Eastern Equine Encephalitis Coinfection With California Serogroup Encephalitis Virus

**DOI:** 10.3389/fneur.2019.00242

**Published:** 2019-03-19

**Authors:** Jonathan J. Cho, Joshua K. Wong, Jacqueline Henkel, Reordan O. DeJesus, Bernadette Nazario-Lopez

**Affiliations:** ^1^Department of Neurology, McKnight Brain Institute, College of Medicine, University of Florida, Gainesville, FL, United States; ^2^Department of Anatomy and Cell Biology, College of Medicine, University of Florida, Gainesville, FL, United States; ^3^Department of Radiology, University of Florida, Gainesville, FL, United States

**Keywords:** Eastern equine encephalitis, IVIg, California encephalitis virus serogroup, seroconversion, coinfection

## Abstract

Eastern equine encephalitis (EEE) is a severe arboviral neuroinvasive disease with high mortality and neurological sequelae. Treatment for EEE is primarily supportive. Intravenous immunoglobulin (IVIg) and high-dose steroids have been used as empirical therapy for EEE with some case reports of benefit. We report a case of a patient who presented with encephalopathy with initial cerebrospinal spinal fluid (CSF) serology analysis showing California serogroup encephalitis virus IgG positivity. However, the rapid clinical deterioration of the patient into a comatose state prompted a second CSF serology analysis that showed seroconversion of high titer Eastern Equine Encephalitis virus IgM and positive titer of California serogroup encephalitis virus IgG. The patient completed a 5-day course of empiric IVIg without concurrent corticosteroid therapy but did not show significant clinical improvement.

## Introduction

Eastern equine encephalitis (EEE) is the most severe arboviral disease with >35% mortality in humans, especially in the <15 and >50 year-old populations (1–4). Eastern equine encephalitis virus (EEEV) is a member of *Togaviridae* family with positive-sense, single-stranded RNA genome (5). The EEEV life cycle is maintained in an enzootic cycle between *Culiseta melanura* mosquitoes that breed in freshwater hardwood swamp areas and birds (4). From 2007–2016, a total of 68 cases of neuroinvasive EEE were reported in the United States with an average of 7 cases per year (6). A recent study implicated Florida in a source-sink model as the major source of EEEV in eastern United States (5). Enzootic EEEV infection has also recently been reported in Panama (7). Mammalian infection of EEEV requires bridging mosquitoes that feed on both birds and mammals, such as the *Aedes* or *Coquillettidia* species (4). EEEV-infected humans typically do not develop a high enough viremia level to allow virus transmission to feeding mosquitoes, thus humans are considered dead-end hosts (4).

Most EEEV infections have no clinical symptoms and < 5% of people infected with EEEV develop viral meningitis or encephalitis (1–4). Acute onset of fever, chills, malaise, myalgia, and arthralgia characterizes systemic EEEV infection (4). Neuroinvasive EEE is characterized by fever, headache, encephalopathy, and seizures (1). In addition to its high mortality rate, EEE results in neurologic sequelae in >50% of survivors (1). Neuroimaging of EEE cases typically show abnormal MRI T2 weighted hyperintensities in the bilateral basal ganglia and thalami with associated restricted diffusion (1, 8). Meningeal enhancement is also frequently present. The diagnosis of EEE is made in the presence of positive EEEV IgM in the CSF (1). Intravenous immunoglobulin (IVIg) and high-dose IV methylprednisolone have been given in several isolated cases reported to have good outcomes, but the relationship between these patient outcomes and these interventions is unknown (1, 9–11).

The California serogroup virus is in the family *Bunyaviridae*, which includes the La Crosse virus (LACV), and is the most common cause of severe symptomatic disease in the pediatric population (2, 6, 12, 13). Clinical symptoms of La Crosse virus infection include fever, headache, vomiting, meningitis, encephalitis, and meningoencephalitis (12, 14). Mortality rate of La Crosse encephalitis is very low (<1%) and most patients recover completely (14). Diagnosis of La Crosse virus is made in the presence of La Crosse virus IgM (14).

## Case Presentation

**Figure 1:**
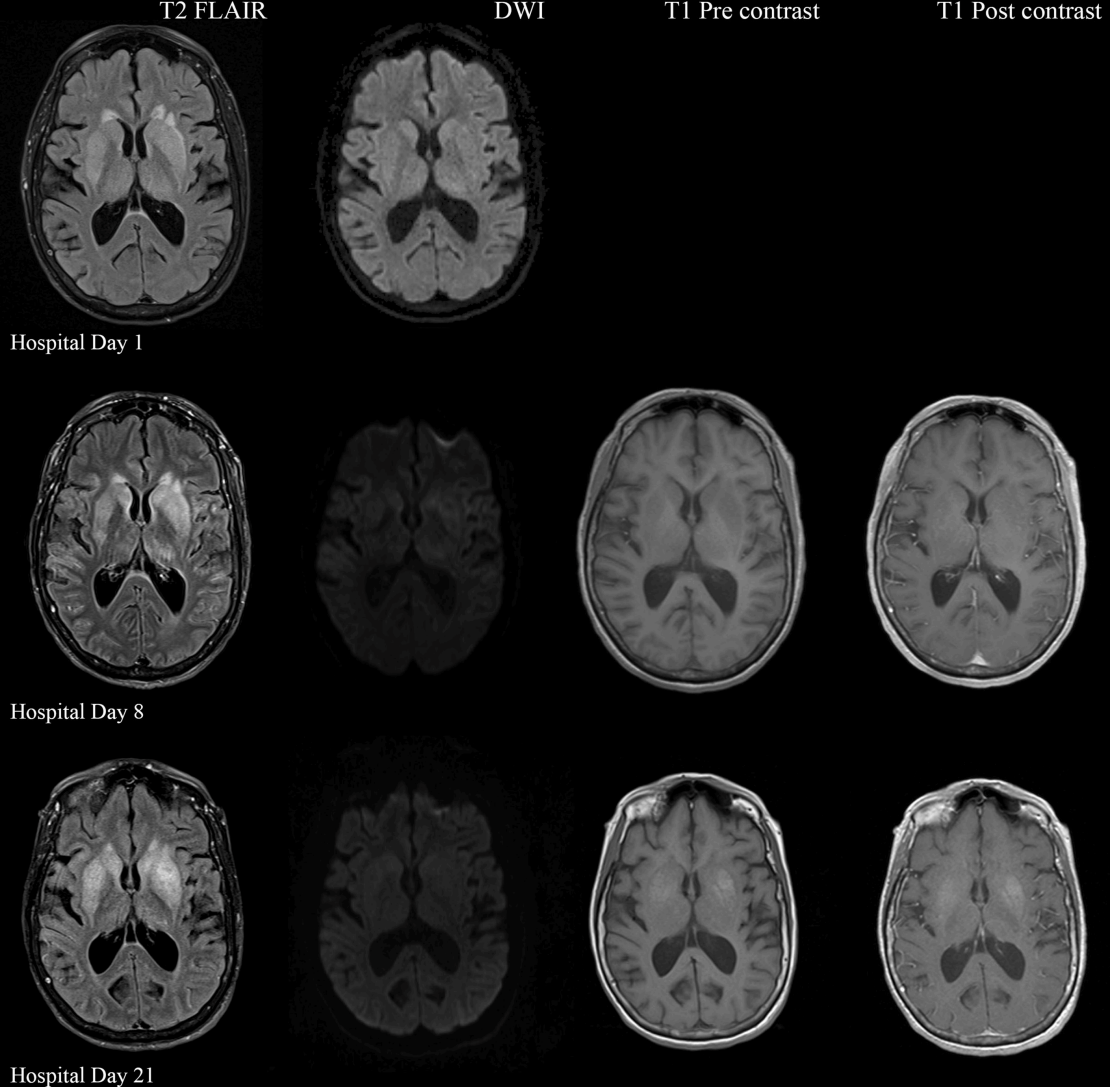
Serial MRI Brain sequences for hospital day 1, 8, and 21. On hospital day 1, there is abnormal hyperintense T2 FLAIR signal in the bilateral deep gray nuclei without associated restricted diffusion. On hospital day 8, there is progressively increasing abnormal T2 signal in the deep gray nuclei as well as restricted diffusion within the areas of abnormal T2 signal. There are no abnormalities on T1 pre and post contrast sequences. On hospital day 21, there is evolving T2 hyperintense signal in the bilateral deep gray nuclei with similar degree of restricted diffusion. There is now an abnormal T1 hyperintense signal in the deep gray nuclei without contrast enhancement, suggestive of developing gliosis.

A 57-year-old woman with a past medical history of hypertension, scoliosis and depression presented with 1 week of flu-like symptoms (diffuse body aches, fatigue, and chills) and generalized weakness that rapidly progressed over several days in June 2018. Her social history is significant for living in a trailer in a wooded area in Florida, living in areas close to a mix of salt and river water, and exposure to mold, rats, and bat droppings near her home. On initial neurological examination, the patient was lethargic but oriented to person, place and time. She had impaired concentration and required occasional tactile or auditory stimulus to maintain attention. There were no cranial nerve deficits. Strength was 4/5 in the bilateral upper extremities and 3/5 in the bilateral lower extremities. There were no pathologic reflexes and sensation to light touch was intact in all extremities. The patient then had a rapid deterioration requiring intubation and mechanical ventilation. Serum laboratory results and serology testing throughout the hospital course are presented in Tables 1–6. Ceftriaxone, acyclovir, vancomycin, and ampicillin were started due to suspicion of meningitis based on the initial clinical presentation and serum testing. Computed Tomography (CT) angiogram of the head was unremarkable. Initial MRI Brain without contrast (hospital day 1) showed abnormally increased T2 FLAIR signal in the deep gray nuclei bilaterally, most prominent in the striatum (Figure 1). Blood cultures were negative throughout her hospitalization. Cerebrospinal fluid (CSF) analysis was suggestive of meningitis and is presented in Table 5. Her CSF arboviral antibody panel is shown in Table 6 and was initially unremarkable except for California encephalitis IgG 1:256.

**Table 1:** Urine drug screen.

	**Ref. range**	**Hospital day 1**
Amphetamine	Range: None detected	None detected
Barbiturate	Range: None detected	None detected
Benzodiazepine	Range: None detected	None detected
Cannabinoid	Range: None detected	None detected
Cocaine metabolites	Range: None detected	None detected
Methadone screen	Range: None detected	None detected
Opiate screen	Range: None detected	None detected
Oxycodone	Range: None detected	None detected
Ethyl alcohol	Range: Not detected	Below detectable limits

**Table 2:** Serum metabolic panel.

	**Ref. range**	**Hospital day 1**	**Hospital day 4**	**Hospital day 8**	**Hospital day 22**
Sodium	Range: 136–145 mmol/L	137	136	146 (H)	139
Potassium	Range: 3.3–5.1 mmol/L	3.6	4.6	3.7	3.4
Chloride	Range: 98–107 mmol/L	103	109 (H)	111 (H)	102
Carbon dioxide	Range: 22–30 mmol/L	23	19 (L)	26	30
Urea nitrogen	Range: 6–21 mg/dL	12	18	14	17
Creatinine	Range: 0.38–1.02 mg/dL	0.87	0.54	0.34 (L)	0.43
Glucose	Range: 65–99 mg/dL	137 (H)	100 (H)	124 (H)	116 (H)
Calcium	Range: 8.4–10.2 mg/dL	8.8	8.6	8.4	9.3
Phosphorus	Range: 2.7–4.5 mg/dL	3.3	3.6	2.6 (L)	2.8
Magnesium	Range: 1.5–2.8 mg/dL	2.0	2.3	2.2	2.1
Total protein	Range: 6.4–8.3 g/dL	7.0			
Albumin	Range: 3.5–5.2 g/dL	4.1			
Albumin/globulin ratio	Latest units: (calc)	1.4			
AST	Range: 0–37 IU/L	24			
ALT (SGPT)	Range: 0–35 IU/L	12			
Bilirubin, direct	Range: 0.0–0.2 mg/dL	< 0.1			
Total bilirubin	Range: 0.0–1.0 mg/dL	0.4			
Alkaline phosphatase	Range: 33–133 IU/L	60			

**Table 3:** Complete blood counts, C-reactive protein.

	**Ref. range**	**Hospital day 1**	**Hospital day 4**	**Hospital day 8**	**Hospital day 22**
WBC	Range: 4.0–10.0 thou/cu mm	14.9 (H)	11.9 (H)	4.9	8.1
RBC	Range: 4.00–5.20 x10E6/uL	4.97	4.22	3.51 (L)	3.81 (L)
Hemoglobin	Range: 12.0–16.0 g/dL	14.9	12.8	10.8 (L)	11.6 (L)
Hematocrit	Range: 35.0–45.0%	43.9	37.5	31.2 (L)	34.2 (L)
MCV	Range: 78.0–100.0 fl	88.3	88.9	89.0	89.7
MCH	Range: 26.0–34.0 pg	29.9	30.2	30.7	30.4
MCHC	Range: 31.0–37.0 g/dL	33.9	34.0	34.4	33.9
RDW	Range: 11.0–14.0%	13.3	13.7	13.5	13.6
Platelet count	Range: 150–450 thou/cu mm	181	193	243	491 (H)
MPV	Range: 6.0–10.0 fl	8.2	9.2	8.3	8.4
Neutrophils	Range: 40.0–80.0%	90.1 (H)	86.4 (H)	64.0	66.8
Lymphocytes	Range: 20.0–45.0%	4.9 (L)	6.2 (L)	20.2	15.1 (L)
Monocytes	Range: 2.0–10.0%	4.6	7.2	14.1 (H)	15.9 (H)
Eosinophils	Range: 0.0–8.0%	0.0	0.0	1.5	1.0
Basophils	Range: 0.0–2.0%	0.4	0.2	0.2	1.2
CRP, high sensitivity	Range: 0.00–5.00 mg/L	10.96 (H)			

**Table 4:** CSF and urine infectious serology panel.

	**Ref. range**	**Hospital day 2**
Cryptococcal Ag CSF	Range: Negative	Not detected
Histoplasma antigen urine	Latest Units: ng/mL	Not detected
Histoplasma galactomannan Ag urine	Range: Not detected	Not detected
EBV quant interpretation CSF	Range: Not detected	Not detected
Enterovirus PCR CSF	Range: Negative	Negative
HSV DNA PCR CSF	Range: Not detected	Not detected
Varicella zoster DNA PCR CSF	Range: Not detected	Not detected

**Table 5:** Cerebrospinal fluid results.

	**Ref. range**	**Hospital day 2**	**Hospital day 7**
WBC	Range: 0– < 5 cu mm	810 (H)	8 (H)
RBC	Range: 0– < 5 cu mm	2	90 (H)
Protein	Range: 15–45 mg/dL	117 (H)	163 (H)
Glucose	Range: 40–70 mg/dL	81 (H)	66
Appearance		Hazy	Clear
Lymphs		19% (H)	83% (H)
Mono/macrocyte		8% (H)	17% (H)
Polys		73% (H)	
Xanthochromia		Clear	Xanthochromia absent
Tube number		3	

**Table 6:** Arbovirus serology panel.

	**Ref. range**	**Hospital day 2**	**Hospital day 7**
California Encephalitis IgG	Range: < 1:16	1:256 (H)	1:64 (H)
California Encephalitis IgM	Range: < 1:16	< 1:16	< 1:16
East Equine IgG	Range: < 1:16	< 1:16	< 1:16
East Equine IgM	Range: < 1:16	< 1:16	1:512 (H)
St Louis IgG	Range: < 1:16	< 1:16	< 1:16
St Louis IgM	Range: < 1:16	< 1:16	< 1:16
West Equine IgG	Range: < 1:16	< 1:16	< 1:16
West Equine IgM	Range: < 1:16	< 1:16	< 1:16
West Nile IgG	Range: ≤ 1.29 IV	0.17	0.17
West Nile IgM	Range: ≤ 0.89 IV	0.02	0.00

Electroencephalogram (EEG) showed diffuse background slowing and rare left temporal sharp waves. On hospital day 4, neurological exam in the setting of pharmacologic sedation and mechanical ventilation was significant for a comatose state with absent brainstem reflexes except for bilateral positive corneal reflexes. She withdrew to painful stimuli in the upper extremities but not lower extremities. Continuous EEG (cEEG) eventually found evidence of non-convulsive status epilepticus (NCSE) and the patient was stabilized with levetiracetam, valproic acid, and lacosamide. Acute viral encephalitis was suspected and a 5-day course of 0.4 g/kg/day IVIg was started on hospital day 7. Results from repeat CSF arbovirus panel drawn 5 days after the first panel (hospital day 7) showed strongly positive EEE IgM titer at 1:512, and positive California encephalitis IgG at 1:64. Follow up MRI Brain with and without contrast (hospital day 8) demonstrated progressive abnormal increased T2 signal in the deep gray nuclei with restricted diffusion. There was new diffuse involvement of the cortex, most prominent in the bilateral mesial temporal lobes and the high convexities. There was also leptomeningeal and perivascular enhancement throughout the cerebral hemispheres. After completion of empirical IVIg trial, the patient continued to be in a comatose state in the absence of pharmacologic sedation. MRI Brain with and without contrast 10 days after completing IVIg (hospital day 22) showed decreased leptomeningeal and pial enhancement suggestive of decreased inflammation. There was again progressive T2 hyperintensity in the deep gray nuclei, with near complete involvement of the caudate and putamen. There were no new areas of restricted diffusion. The patient was successfully weaned off mechanical ventilation 5 weeks after empiric IVIg treatment completion. During clinic follow-up 6 weeks post-IVIg trial completion, neurological exam showed an awake state with spontaneous eye opening, presence of bilateral blink to threat, intact corneal reflexes, and withdrawal to noxious stimuli in all extremities. She was non-verbal, unable to follow simple instructions, unable to walk, and completely dependent in her activities of daily living.

## Discussion

Recent case reports of possible positive outcome from empiric IVIg and high-dose IV methylprednisolone (1 g/day) in EEE suggest that a part of the neuropathology associated with EEEV infection may be due to the inflammatory reaction in the brain. EEE patients treated with IVIg were able to follow commands within 1–6 days after IVIg initiation (9–11). Many factors including timing of IVIg administration during EEE disease course may contribute to the decreased effectiveness of IVIg. Various mechanisms underlying the immunomodulatory effect of IVIg have been suggested (15). The dimeric antigen-binding fragment, F(ab')_2_, and the constant fragment (Fc), have both been shown to be crucial for immunosuppression in IVIg therapy (15). F(ab')2-dependent mechanisms involves inflammatory cell depletion, cellular receptors blockade, cytokines and autoantibody neutralization, and anaphylatoxin scavenging (15). Saturating the neonatal Fc receptor (FcRn), promoting expansion of regulatory T (Treg) cells, blocking activating receptor, modulating dendritic cells, blocking immune complex binding to low-affinity Fcγ receptors (FcγRs), and modulation of activating and inhibitory FcγR expression on innate immune effector cells and B cells are Fc-dependent mechanisms attributed to IVIg (15).

The clinical manifestations of multiple arbovirus infections in a single patient have not been well-characterized. This case report showed an acute seroconversion of EEEV IgM in the background of the patient's California serogroup encephalitis virus IgG seropositivity, the patient remained in a comatose state upon completion of IVIg therapy. She may have experienced a more severe encephalitis due to neuroinvasive EEEV coinfection on California serogroup encephalitis virus infection. Recent studies have shown that the La Crosse virus induces neuronal death through activation of innate immune signaling (16). EEEV was recently shown to avoid innate immune activation by using a host microRNA to restrict viral replication in innate immune cells, and the degree of peripheral innate immune escape is directly correlated to mortality in mice (17). Concomitant innate immune signaling activation by a California serogroup encephalitis virus and peripheral innate immune escape of EEEV in the current patient may explain the rapid progression to comatose state and decreased effectiveness of IVIg treatment.

In this patient, initial CSF serology testing showed only California serogroup encephalitis virus IgG positivity. The rapidly deteriorating clinical course of a patient presenting with encephalopathy and then progressing to comatose state within 1 day is atypical for California serogroup encephalitis, especially in adults (13, 14). However, a case of encephalitis and rapid cognitive decline associated with Jamestown Canyon virus, a member of the California serogroup viruses, was reported in a 73-year-old male (18). This raises the possibility of attributing the patient's severe clinical presentation to coinfection of EEEV and Jamestown Canyon virus as arbovirus coinfection can augment *in vivo* replication [reviewed in Vogels et al. (19)]. Repeat CSF serology 5 days later revealed high EEEV IgM titer along with California serogroup encephalitis virus IgG. Indirect immunofluorescence assay for Rabies virus is known to have cross-reactivity with West Nile and Powassan flaviviruses (20), which raises the possibility that our detection of California serogroup encephalitis virus and EEEV could be due to cross-reactivity. However, cross-reactivity is unlikely because we detected California serogroup encephalitis virus IgG initially without EEEV IgM or IgG and EEEV IgM was only detected in the repeat CSF serology 5 days later, showing acute seroconversion of EEEV. If cross-reactivity did occur, then both EEEV IgM or IgG would be detected concomitantly with California serogroup encephalitis virus IgM or IgG in both CSF samples. Another limitation of this case report is the lack of PCR study confirming the presence of EEEV and California serogroup encephalitis virus in the patient's CSF. Only 3 cases of EEE with good outcomes have been reported in the setting of empiric IVIg therapy (9–11). No randomized clinical trials have examined the efficacy of IVIg in EEE, likely due to the rarity of EEE (6).

This report presents a unique case of acute EEEV seroconversion with coinfection on California serogroup encephalitis virus infection. Repeat CSF serology testing should be performed to confirm a clinical suspicion of EEE. IVIg therapy can be considered if the clinical suspicion of EEE is high though blinded randomized controlled trials are needed to establish whether IVIg has any efficacy in EEEV encephalitis. Additional research is needed to elucidate the mechanisms underlying the effect of IVIg in EEE and the disruption of such therapeutic mechanisms in the presence of multiple arbovirus disease processes.

## Ethics Statement

This case study was exempted due to no change in standard of care. Informed consent for publication was obtained from patient's next-of-kin.

## Author Contributions

JC: medical student; JW: resident physician; BN-L: neurology attending physician on the management team of the patient; JH and RD: interpreted radiological findings and prepared figures and legends; JC, JW, JH, RD, and BN-L: wrote the manuscript.

### Conflict of Interest Statement

The authors declare that the research was conducted in the absence of any commercial or financial relationships that could be construed as a potential conflict of interest.
